# Pressure Overload by Transverse Aortic Constriction Induces Maladaptive Hypertrophy in a Titin-Truncated Mouse Model

**DOI:** 10.1155/2015/163564

**Published:** 2015-10-04

**Authors:** Qifeng Zhou, Scott Kesteven, Jianxin Wu, Parwez Aidery, Meinrad Gawaz, Michael Gramlich, Michael P. Feneley, Richard P. Harvey

**Affiliations:** ^1^Department of Cardiology and Cardiovascular Diseases, Eberhard Karls University, 72076 Tübingen, Germany; ^2^Victor Chang Cardiac Research Institute, Darlinghurst, NSW 2010, Australia; ^3^St. Vincent's Clinical School, University of New South Wales, Kensington, NSW 2010, Australia; ^4^Cardiology Department, St. Vincent's Hospital, Darlinghurst, NSW 2010, Australia; ^5^School of Biological and Biomolecular Sciences, University of New South Wales, Kensington, NSW 2010, Australia

## Abstract

Mutations in the giant sarcomeric protein titin (TTN) are a major cause for inherited forms of dilated cardiomyopathy (DCM). We have previously developed a mouse model that imitates a TTN truncation mutation we found in a large pedigree with DCM. While heterozygous *Ttn* knock-in mice do not display signs of heart failure under sedentary conditions, they recapitulate the human phenotype when exposed to the pharmacological stressor angiotensin II or isoproterenol. In this study we investigated the effects of pressure overload by transverse aortic constriction (TAC) in heterozygous (Het) *Ttn* knock-in mice. Two weeks after TAC, Het mice developed marked impairment of left ventricular ejection fraction (*p* < 0.05), while wild-type (WT) TAC mice did not. Het mice also trended toward increased ventricular end diastolic pressure and volume compared to WT littermates. We found an increase in histologically diffuse cardiac fibrosis in Het compared to WT in TAC mice. This study shows that a pattern of DCM can be induced by TAC-mediated pressure overload in a TTN-truncated mouse model. This model enlarges our arsenal of cardiac disease models, adding a valuable tool to understand cardiac pathophysiological remodeling processes and to develop therapeutic approaches to combat heart failure.

## 1. Introduction

Dilated cardiomyopathy (DCM), a heart disease that is characterized by left ventricular dilatation, reduction in left ventricular function, and occurrence of cardiac arrhythmias, is a major cause for congestive heart failure [1]. About 20–48% of DCM cases are inherited with mutations in a variety of genes encoding sarcomeric, cytoskeletal, and nuclear membrane proteins, as well as proteins involved in Ca^2+^ metabolism [2].

The sarcomeric protein titin (TTN) is the biggest known single-copy protein in humans, mainly expressed in muscle tissue [3]. A single TTN molecule spans half the sarcomere and links the Z-disc with the M-line. As a pivotal building block of the sarcomere, it provides passive forces and mainly contributes to the elasticity of a muscle [4]. TTN also plays a major role in scaffolding and coordinating structural and signal proteins for mechanotransduction [5].

TTN mutations were linked to DCM more than a decade ago [6]. Recently, TTN truncating mutations emerged as the leading genetic cause of DCM in human patients, accounting for about 25% of cases of familial DCM and 18% for idiopathic DCM [7]. TTN truncating mutations are not randomly distributed in this big protein but are predominantly presented at the A-band region [8].

We have previously generated a mouse model that imitates a human truncation mutation we found in a large DCM pedigree. The 2 bp insertion is located in exon 326 of titin, resulting in a premature stop codon with stop of translation in A-band TTN [6].

While the homozygous TTN knock-in mice die* in utero* on about day 9.0 of gestation due to severe defects in sarcomeric assembly, the Het animals do not develop a cardiac phenotype under sedentary conditions. However, when exposed to pharmacological stress (e.g., angiotensin II or isoproterenol), they show all features of DCM and therefore recapitulate the human disease [9].

Efforts to dissect pathways that are involved in DCM development and progression in this mouse model have failed so far, since the pharmacological agents (e.g., angiotensin) enormously disturbed expression levels of TTN binding partners, making it impossible to trace subtle differences induced by the TTN mutation [9]. To omit this, we attempted the use of aortic constriction as a mechanical stressor to induce DCM in our mouse model.

## 2. Materials and Methods

### 2.1. TTN Knock-In Mice

The TTN knock-in mouse line, harbouring a 2 bp insertion mutation in A-band TTN, was described previously [9]. Briefly, the mutation was introduced into mouse embryonic stem cells by homologous recombination with a plasmid carrying the mutation with flanking genomic DNA sequences, followed by antibiotic selection of ES cell clones, blastocyst injections, and embryo transfer. Pups were tested for chimerism and the mice were evaluated for transmission of the mutation to their offspring. Het mice were back-crossed to a C57Bl/6 background for at least 9 generations. All animal investigations were approved by the Institutional Animal Care and Use Committee, as well as the local Animal Review Board.

### 2.2. Transverse Aortic Constriction on Mouse

Mice at the age of 3-4 months were anesthetized with ketamine (100 mg/kg) and xylazine (10 mg/kg) and transferred to a heated platform. Anesthetized mice were intubated, followed by midline cervical incision to expose the aorta. Aortic constriction was achieved by placing a 7.0 nylon suture ligature against a 27-gauge needle on transverse aorta. The needle was removed promptly to create an aortic constriction of 0.4 mm in diameter and the chest was sealed. In Sham operated mice, the procedure was the same besides the constriction of aorta. The mice were maintained on a heating pad for recovery.

### 2.3. Echocardiography

Echocardiography was performed as described previously [9]. Briefly, anesthesia was induced with 4-5% isoflurane in an anesthetic chamber; mice were then restrained supine on a heated platform. Anesthesia was maintained with 0.5–1.5% isoflurane; body temperature was kept at 37°C. An ultrasound system (Vevo 770, VisualSonics, Toronto, ON, Canada) was used for the echocardiographic analysis with a 30-MHz probe. Images were acquired and stored as a digital cine loop for offline calculations. Standard imaging planes, M-mode, Doppler, and functional calculations were obtained according to the American Society of Echocardiography guidelines. M-mode derived from parasternal short-axis view of the left ventricle recorded at 1 kHz (EKV) was used to determine wall thickness, end systolic and end diastolic diameters, ventricular dimensions and volumes, and ejection fraction.

### 2.4. Left Ventricular Hemodynamics

On day 14 after TAC surgery, hemodynamic measurements were obtained. Mice were anesthetized with isoflurane and transferred to a heated platform where a nose cone delivered isofurane (0.5–2.0%). A micromanometer-tipped catheter (Millar Inc., Texas, US) was introduced into the left ventricle through the right carotid artery. The transducer was connected to an external recorder and aortic pressure and ventricular pressure were recorded at 1 kHz (BIOPAC Inc., CA, US). Immediately after removal of the catheter mice were sacrificed and heart tissue was obtained for histological analysis.

### 2.5. Masson's Trichrome Staining

Heart tissue was fixed with 4% PFA, dehydrated in serial graded ethanol (70%, 80%, and 90%, each for 1 hour and twice in 100% ethanol for 1 hour), and embedded into paraffin blocks. The tissue blocks were sectioned with a Leica Microtome and transferred to glass slides. To remove paraffin, the slides went through serial graded ethanol (100%, 90%, 80%, and 70%, each for 5 minutes) and rehydrated in H_2_O for 5 minutes. Sections (8 *μ*m) were stained with Masson's trichrome to detect fibrosis. Quantification of fibrosis was performed using an image analysis system (ImageJ).

### 2.6. Statistics

Within-group statistical comparisons for each genotype were made with ANOVA for repeated measures. Between-group comparisons were made with two-way ANOVA. Dunnett's multiple comparison test was used to isolate the source of difference. Data are reported as mean ± SEM. Values of *p* < 0.05 were considered significant.

## 3. Results

### 3.1. TAC-Mediated Pressure Overload Induced Maladaptive Left Ventricular Hypertrophy and Impaired Systolic Function in Heterozygous* Ttn* Knock-In Mice

We performed TAC banding in heterozygous* Ttn* knock-in mice and wild-type littermate controls. As an additional control, an equally sized Sham group (heterozygous and wild-type) was used.

**Figure 1:**
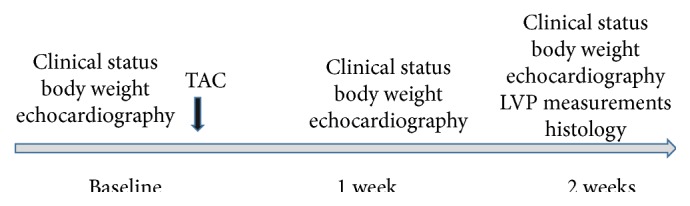
Schematic of the study design.

The study was designed as shown in Figure 1. Prior to surgery, clinical status and body weight were assessed in all mice. In addition, heart function and morphology were determined by echocardiography. On the same day, thoracic aortic constriction (TAC) surgery was performed by an experienced operator. Follow-up clinical assessment and echocardiography were obtained on day 7 and day 14 after TAC. On day 14 after TAC, left ventricular and aortic pressure loops were recorded, the mice were sacrificed, and the heart was processed for histology studies.

**Figure 2:**
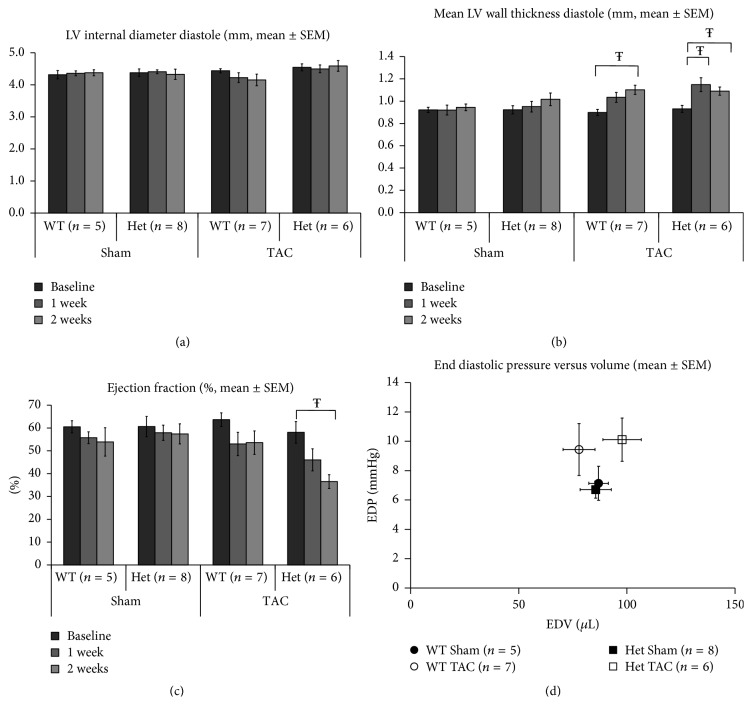
Echocardiographic and LV pressure assessments of WT and Het mice at baseline, week 1, and week 2 following TAC. (a) There is no evidence of LV dilatation in either genotype in either the presence or absence of TAC. (b) The onset of ventricular hypertrophy appears to be more rapid in the Het group demonstrating significant increases by week 1 unlike WT mice which only manifest significant thickening by week 2. (c) The reduction in EF% induced by TAC in the Het group is significant by week 2, unlike the WT TAC mice which tended to demonstrate a fall in this measure of contractile function but did not reach significance at either time point after TAC surgery. (d) Differences in EDP between groups do not reach significance although the trend is towards increase in both TAC groups. Interestingly the EDV trend although also not significant for the Het TAC group moves to the right of the Sham Het group, a change reflective of ventricular dilatation, whereas the EDV of the WT TAC mice changes little and even moves to the left more aligned to a physiological adaptation (^Ŧ^ *p* < 0.05). Sample sizes are also shown.

As reported previously [9], there were no differences in left ventricular diameters, left ventricular end diastolic dimension (LVEDD) and left ventricular end systolic dimension (LVESD), ventricular wall thicknesses (interventricular septum in diastole, IVSd), and cardiac contractility (ejection fraction, EF) between Het* Ttn* knock-in mice and their WT littermates at baseline (Figure 2). One week after TAC signs of cardiac hypertrophy were apparent with significant increases in LV mean wall thickness in diastole (LVMWd) only in the TTN group (LVMWd; Het; baseline 0.93 ± 0.03 mm versus wk1 1.15 ± 0.06 mm; *p* < 0.05). At the same time point no significant changes in left ventricular end diastolic diameters or EDV were noted in Het mice (LVEDd; WT: Sham 4.36 ± 0.07 mm versus TAC 4.39 ± 0.21 mm, Het Sham 4.41 ± 0.06 mm versus TAC 4.50 ± 0.12 mm). TAC induced increased wall thickness in both WT and Het mice but this increase from baseline was statistically significant at both week 1 and week 2 in Het mice whereas it was significant only at week 2 in WT mice (LVMWd: WT; baseline 0.92 ± 0.03 mm versus wk2 1.10 ± 0.04 mm, Het; baseline 0.93 ± 0.03 mm versus wk2 1.09 ± 0.04 mm; *p* < 0.05). Strikingly, contractile cardiac function (EF%) demonstrated a continuing fall in the Het TAC group from 58 ± 5% at baseline to 46 ± 5% at week 1 and to 37 ± 3% at week 2 (*p* < 0.05), whereas in WT animals falls in EF% did not reach significance from baseline to the first or second week (WT; baseline 64 ± 3%, wk1 53 ± 5%, wk2 54 ± 5, Figure 2).

The Sham operated mice did not show signs of cardiac remodeling in both the WT and Het group animals, and there was no impairment of cardiac function until the experiment was terminated. A complete list with all echocardiographic data is available in Supplementary Figure 1 (see Supplementary Material available online at http://dx.doi.org/10.1155/2015/163564).

### 3.2. Alterations of Hemodynamic Parameters Mediated by TAC

Two weeks after TAC banding, cardiac catheterization was performed and left ventricular pressure as well as aortic pressure was recorded (Figure 2).

Two weeks after TAC, systolic aortic pressure (AoPs) increased in the TAC groups compared to their corresponding Sham operated controls, respectively (WT TAC, 180 ± 6.9 mmHg versus WT Sham, 128 ± 11 mmHg; Het TAC, 164 ± 5 mmHg versus Het Sham, 116 ± 3 mmHg, *p* < 0.01), demonstrating successfully performed aortic constriction. There was no significant difference in AoPs between the WT TAC group and the Het TAC group. We also found no significant differences in diastolic aortic pressure (AoPd) between Het and WT animals. Two weeks after TAC, left ventricular systolic pressure (LVSP) was increased in the TAC groups compared to their Sham operated controls (WT TAC, 181 ± 7 mmHg versus WT Sham, 130 ± 7 mmHg; Het TAC, 168 ± 4 mmHg versus Het Sham, 115 ± 4 mmHg, *p* < 0.01). Interestingly, TAC banding induced a trend to higher left ventricular end diastolic pressure (LVEDP) in the Het TAC group compared to Het Sham group and WT TAC compared to WT Sham groups (Het TAC, 10.1 ± 1.5 mmHg versus Het Sham, 6.7 ± 0.6 mmHg, or WT TAC, 9.4 ± 1.8 mmHg versus WT Sham, 7.1 ± 1.2). Heart rate during LV measurements was as follows: WT Sham 457 ± 14 bpm; Het Sham 441 ± 37 bpm; WT TAC 437 ± 61 bpm; Het TAC 498 ± 90 bpm (n.s. between groups).

### 3.3. Cardiac Fibrosis in TTN A-Band Truncating Mutation Knock-In Mice

**Figure 3:**
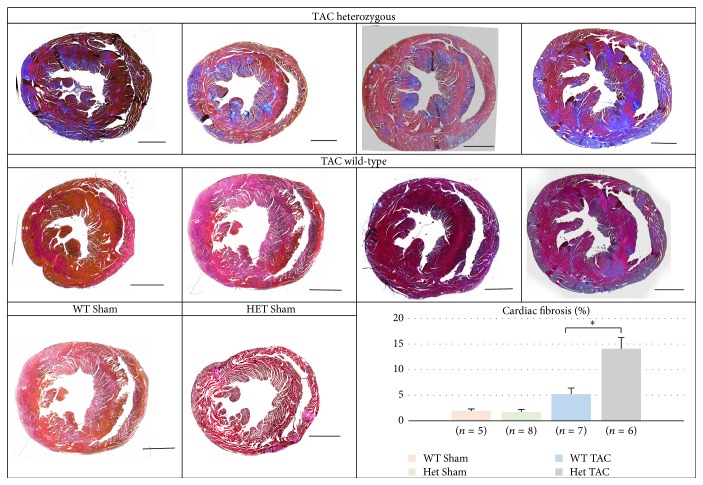
Myocardial histology of 4 representative heart sections from wild-type and Het animals 2 weeks after TAC, stained with Masson's trichrome. One representative Sham heart per genotype is also shown. Note the increased level of fibrosis in Het hearts (^*∗*^ *p* < 0.01) compared to their WT littermates. Bar = 1 mm.

Cardiac extracellular matrix (ECM) remodeling is a process adapted by the heart to deal with physiological and pathophysiological hemodynamic changes. Excess ECM protein production and deposition in the myocardium (cardiac fibrosis) is a hallmark of maladaptive remodeling in a failing heart. We analyzed cardiac fibrosis in 7 hearts per genotype by Masson's trichrome staining in heart muscle tissue obtained 2 weeks after TAC. While WT mice showed only distinct fibrotic areas, heterozygous animals developed massive cardiac fibrosis (5.2 ± 1.5% versus 14.1 ± 4%, *p* < 0.01) (Figure 3). There was no cardiac fibrosis detectable in Sham operated mice.

## 4. Discussion

In this study, we showed that pressure overload by thoracic aortic constriction induces maladaptive hypertrophy with impaired left ventricular function in a mouse model with a TTN truncation mutation we found in a family with dilated cardiomyopathy.

TTN truncating variants have been described as the major disease gene for dilated cardiomyopathy, accounting for approximately 25% of all cases [7]. However, TTN truncations can also be found in the healthy population, which gives some uncertainty to the pathogenic value of such variants. Recently, Roberts et al. [8] used genetic, transcriptome, and protein information to differentiate between polymorphisms and true disease causing mutations. This landmark study gave strong evidence that truncation mutations located in A-band TTN are most commonly disease causing, whereas I-band variants can generally be well compensated by the organism. Our mouse model harbors a truncation variant in A-band TTN, making it a good mouse model for a wide spectrum of human DCM. In addition, our data strongly support the evidence of a pathogenetic role for A-band variants.

As described previously, heterozygous* Ttn* knock-in mice do not develop a cardiac phenotype under resting conditions [9]. Interestingly, disease penetrance in the family with the corresponding mutation [6], as well as in most other described DCM families, is incomplete, indicating that a “second hit” (environmental or a second genetic modifier) is required for disease development and progression. The need of a cardiac stressor in our mouse model (here: pressure overload by TAC) supports the “second hit” hypothesis in titin-based DCM.

TTN is a major contributor of cardiac stiffness. We have previously performed active and passive tension measurements in skinned ventricular papillary muscle fibers as well as echocardiographic evaluation of diastolic function in our Het* Ttn* mouse model [9]. We did not find any changes in viscoelastic properties in these animals. This is most likely because the predominant mechanism of disease in our model is haploinsufficiency, with degradation of the mutant protein and compensatory upregulation of the wild-type protein. Under the hypertrophic stimulus of pressure overload, this compensatory mechanism seems to fail, resulting in a lack of sarcomeric TTN. Presumably, the shortage of the sarcomeric “ruler” TTN results in cardiac disarray with fibrosis and impaired systolic function.

Cardiac extracellular matrix (ECM) remodeling is an adaptive process to deal with physiological and pathophysiological hemodynamic changes [10]. Excess ECM protein production and deposition in the myocardium (cardiac fibrosis) is a hallmark of maladaptive remodeling [11, 12]. A-band TTN truncation mutations produce a shorter TTN protein with impaired M-band signaling. TTN M-band, one of the “hot spots” for mechanotransduction in the sarcomere, contributes to pathological cardiac remodeling [13, 14]. For example, DRAL/FHL2 binds to TTN at the M-line [15] and shuttles between the cytoplasm and the nucleus to regulate gene transcription. MURF1, an E3 ubiquitinase, binds to M-band titin and regulates gene expression and protein turnover [16]. TTN kinase (TK) domain associates with nbr1 and p62, which are receptors for the selective degradation of ubiquitinated proteins by the autophagosome [17]. Together, these findings suggest that TTN A-band truncating mutations could possibly result in defected M-band mechanosensing and transduction, which in turn impairs cardiac protein turnover and remodeling. Hyperactivation of cardiac fibrosis could be one of the consequences of impaired mechanotransduction pathways in the TTN A-band truncating mutant mice, which would contribute to systolic dysfunction in those mice under pressure overload.

In previous studies [9, 18], we used pharmacological stress (angiotensin II, isoproterenol) to induce DCM in our mouse model. Since TTN is known to play a role in biomechanical sensing and signalling [19–21], we performed extensive expression studies with known TTN ligands involved in those functions [9], but we could not find any significant changes in our heterozygous mice. However, expression levels of these binding partners were enormously influenced by the pharmacological agent (angiotensin) itself. Therefore, subtle differences between wild-type and Het mice were supposedly below the detection level. In this study, we used a mechanical stressor and could therefore exclude pharmacological confounders. This should enable us to dissect pathways involved in TTN-based DCM and to develop novel therapeutic strategies to combat heart failure.

## 5. Conclusions

In this study, we showed that TAC-mediated pressure overload leads to hemodynamic impairment and rapid cardiac remodeling in heterozygous TTN A-band truncated mice, resulting in a DCM-like phenotype. Since TTN A-band truncating variants are a major cause for human DCM, the mouse model is a useful tool for the elucidation of disease mechanisms and for the development of novel therapeutic interventions.

## Supplementary Material

Detailed echocardiographic data of WT and Het mice at baseline, week 1 and week 2 after thoracic aortic constriction (TAC).

## References

[1] Nichols M., Townsend N., Scarborough P., Rayner M. (2014). Cardiovascular disease in Europe 2014: epidemiological update. *European Heart Journal*.

[2] Morita H., Seidman J., Seidman C. E. (2005). Genetic causes of human heart failure. *The Journal of Clinical Investigation*.

[3] Granzier H. L., Labeit S. (2004). The giant protein titin. A major player in myocardial mechanics, signaling, and disease. *Circulation Research*.

[4] Labeit S., Kolmerer B. (1995). Titins: giant proteins in charge of muscle ultrastructure and elasticity. *Science*.

[5] Linke W. A. (2008). Sense and stretchability: the role of titin and titin-associated proteins in myocardial stress-sensing and mechanical dysfunction. *Cardiovascular Research*.

[6] Gerull B., Gramlich M., Atherton J. (2002). Mutations of TTN, encoding the giant muscle filament titin, cause familial dilated cardiomyopathy. *Nature Genetics*.

[7] Herman D. S., Lam L., Taylor M. R. G. (2012). Truncations of titin causing dilated cardiomyopathy. *The New England Journal of Medicine*.

[8] Roberts A. M., Ware J. S., Herman D. S. (2015). Integrated allelic, transcriptional, and phenomic dissection of the cardiac effects of titin truncations in health and disease. *Science Translational Medicine*.

[9] Gramlich M., Michely B., Krohne C. (2009). Stress-induced dilated cardiomyopathy in a knock-in mouse model mimicking human titin-based disease. *Journal of Molecular and Cellular Cardiology*.

[10] Cohn J. N., Ferrari R., Sharpe N. (2000). Cardiac remodeling—concepts and clinical implications: a consensus paper from an international forum on cardiac remodeling. *Journal of the American College of Cardiology*.

[11] Kehat I., Molkentin J. D. (2010). Molecular pathways underlying cardiac remodeling during pathophysiological stimulation. *Circulation*.

[12] Yoshimura M., Yasue H., Okumura K. (1993). Different secretion patterns of atrial natriuretic peptide and brain natriuretic peptide in patients with congestive heart failure. *Circulation*.

[13] Gotthardt M., Hammer R. E., Hübner N. (2003). Conditional expression of mutant M-line titins results in cardiomyopathy with altered sarcomere structure. *Journal of Biological Chemistry*.

[14] Hoshijima M. (2006). Mechanical stress-strain sensors embedded in cardiac cytoskeleton: Z disk, titin, and associated structures. *The American Journal of Physiology—Heart and Circulatory Physiology*.

[15] Lange S., Auerbach D., McLoughlin P. (2002). Subcellular targeting of metabolic enzymes to titin in heart muscle may be mediated by DRAL/FHL-2. *Journal of Cell Science*.

[16] Witt S. H., Granzier H., Witt C. C., Labeit S. (2005). MURF-1 and MURF-2 target a specific subset of myofibrillar proteins redundantly: towards understanding MURF-dependent muscle ubiquitination. *Journal of Molecular Biology*.

[17] Lamark T., Kirkin V., Dikic I., Johansen T. (2009). NBR1 and p62 as cargo receptors for selective autophagy of ubiquitinated targets. *Cell Cycle*.

[18] Gramlich M., Pane L. S., Zhou Q. F. (2015). Antisense-mediated exon skipping: a therapeutic strategy for titin-based dilated cardiomyopathy. *EMBO Molecular Medicine*.

[19] Granzier H., Labeit S. (2007). Structure-function relations of the giant elastic protein titin in striated and smooth muscle cells. *Muscle and Nerve*.

[20] Miller M. K., Granzier H., Ehler E., Gregorio C. C. (2004). The sensitive giant: the role of titin-based stretch sensing complexes in the heart. *Trends in Cell Biology*.

[21] Lange S., Xiang F., Yakovenko A. (2005). The kinase domain of titin controls muscle gene expression and protein turnover. *Science*.

