# Supplementary material for: Pressure Overload by Transverse Aortic Constriction Induces Maladaptive Hypertrophy in a Titin-Truncated Mouse Model
Source: Biomed Res Int. 2015 Oct 4;2015:163564. doi: 10.1155/2015/163564 (PMC4609346; doi:10.1155/2015/163564)
Supplement: Supplementary file 1 — Detailed echocardiographic data of WT and Het mice at baseline, week 1 and week 2 after thoracic aortic constriction (TAC). [file 163564.f1.pdf]

Echocardiographic Left Ventricular data from Titin WT and Het TAC bar

2 weeks after TAC

WT	Sham

Het	Sham

WT	TAC

Het	TAC

p values

ıded and sham mice

	Weight	HR	IVS;d	LVID;d	LVPW;d	IVS;s
	gm	BPM				
Mean	31.1	420	1.04	4.38	0.85	1.54
SD	2.28	56	0.14	0.21	0.13	0.25
Max	33.2	469	1.21	4.74	1.07	1.95
Median	31.6	438	1.07	4.29	0.80	1.49
Min	27.4	341	0.88	4.23	0.76	1.28
Mean+2SD	35.6	531	1.32	4.80	1.10	2.04
Mean-2SD	26.5	308	0.76	3.96	0.59	1.05

	gm	BPM				
Mean	31.9	406	1.07	4.33	0.96	1.58
SD	2.63	34	0.14	0.45	0.20	0.24
Max	36.0	443	1.20	4.85	1.26	1.86
Median	31.7	417	1.11	4.39	0.94	1.67
Min	28.6	331	0.76	3.60	0.66	1.13
Mean+2SD	37.1	473	1.35	5.23	1.37	2.06
Mean-2SD	26.6	338	0.80	3.42	0.55	1.10

	gm	BPM				
Mean	31.4	417	1.18	4.15	1.03	1.50
SD	2.35	46	0.17	0.47	0.10	0.17
Max	35.2	470	1.41	4.69	1.21	1.78
Median	31.2	422	1.18	4.31	1.02	1.46
Min	28.5	342	0.98	3.27	0.91	1.33
Mean+2SD	36.1	508	1.51	5.10	1.23	1.85
Mean-2SD	26.7	326	0.84	3.20	0.82	1.16

	gm	BPM				
Mean	30.3	458	1.10	4.59	1.08	1.48
SD	2.20	26	0.11	0.41	0.15	0.15
Max	32.0	494	1.28	5.40	1.26	1.67
Median	31.4	462	1.06	4.48	1.11	1.48
Min	26.7	424	0.97	4.31	0.87	1.29
Mean+2SD	34.7	509	1.32	5.40	1.38	1.78
Mean-2SD	25.9	407	0.87	3.78	0.79	1.18
Baseline; WT v	0.58	0.57	0.69	0.83	0.30	0.78
WT; Sham vs T	0.82	0.93	0.18	0.35	0.02	0.76
Het; Sham vs T	0.25	0.01	0.74	0.29	0.23	0.40
TAC; WT vs He	0.40	0.08	0.35	0.11	0.43	0.82

LVID;s	LVPW;s		%LVPW thick	% IVS thick	FS %	LVd Mass Mar
3.14	1.22	0.94	45.1	49.3	28.3	172
0.48	0.23	0.07	25.30	25.10	9.68	25
3.49	1.43	1.01	74.7	82.2	44.0	209
3.37	1.36	0.95	33.6	39.1	26.4	171
2.37	0.96	0.84	21.0	25.6	20.1	141
4.10	1.69	1.08	95.7	99.5	47.7	223
2.19	0.76	0.81	-5.5	-0.9	9.0	121
3.04	1.30	1.02	37.5	47.3	30.5	188
0.63	0.23	0.16	20.69	12.60	8.19	50
4.06	1.54	1.23	70.0	67.6	44.7	292
3.10	1.33	1.02	35.8	46.4	30.4	185
1.99	0.89	0.71	11.0	24.5	15.9	127
4.30	1.77	1.34	78.8	72.5	46.9	289
1.77	0.83	0.70	-3.9	22.1	14.1	87
3.02	1.46	1.10	42.2	28.5	28.0	197
0.67	0.25	0.11	19.54	7.49	9.66	41
3.57	1.74	1.23	78.1	39.8	47.7	257
3.22	1.43	1.10	40.2	29.1	23.9	176
1.71	1.13	0.97	24.2	17.7	19.3	152
4.36	1.95	1.32	81.2	43.5	47.4	278
1.68	0.97	0.88	3.1	13.6	8.7	115
3.77	1.31	1.09	21.5	36.5	17.6	226
0.24	0.16	0.09	8.81	20.61	4.09	30
4.17	1.51	1.22	32.5	72.2	22.8	268
3.72	1.34	1.12	21.2	33.4	18.3	227
3.58	1.11	0.96	8.7	10.3	11.5	179
4.24	1.64	1.27	39.1	77.7	25.8	285
3.30	0.98	0.91	3.9	-4.7	9.4	166
0.76	0.58	0.36	0.56	0.85	0.68	0.52
0.74	0.12	0.02	0.82	0.06	0.96	0.26
0.02	0.91	0.33	0.10	0.24	0.00	0.13
0.03	0.23	0.84	0.04	0.36	0.03	0.18

LVs Mass Mar	LV masses diff	LVwtd / Bdy Wt		ESV teich	EF teich	SV teich
183	10%	5.5		40	54%	47
14	12%	0.72		13	14%	11
207	29%	6.6		51	76%	60
181	4%	5.2		46	52%	48
173	1%	4.9		20	41%	35
211	34%	7.0		67	82%	69
156	-14%	4.1		14	26%	24
189	9%	5.8		38	57%	47
43	6%	1.15		18	12%	8
264	18%	8.1		73	77%	64
193	10%	5.7		38	58%	47
137	0%	4.3		13	34%	37
275	21%	8.1		75	82%	64
104	-3%	3.5		2	33%	31
199	5%	6.3		38	54%	40
50	4%	1.27		16	14%	6
262	13%	7.9		53	80%	49
179	4%	6.2		42	48%	40
133	1%	4.3		9	40%	33
300	13%	8.8		71	81%	51
99	-3%	3.7		5	26%	28
244	9%	7.5		61	37%	37
43	6%	1.45		9	7%	15
308	18%	10.1		77	45%	64
241	9%	7.4		59	38%	33
186	3%	5.7		54	25%	21
329	22%	10.4		80	51%	66
159	-3%	4.6		43	22%	7
0.77	0.79	0.60	0.46	0.83	0.64	0.89
0.51	0.32	0.26	0.27	0.81	0.97	0.19
0.04	0.82	0.03	0.43	0.02	0.00	0.11
0.11	0.17	0.13	0.53	0.01	0.02	0.61

EDV teich	CO teich	LV mass / LVI[	h/r	HR	VTI	Peak Vel
				BPM	cm	mm/s
87	19.7	39.2	0.43	420	4.0	1011
10	5.97	4.36	0.03	53	1.00	237
104	28.2	44.1	0.47	466	5.5	1319
83	20.7	39.1	0.43	436	3.7	951
80	12.2	32.9	0.39	340	3.0	719
107	31.6	47.9	0.49	526	6.0	1484
67	7.7	30.4	0.37	315	2.0	538

				Doppler data		
				BPM	cm	mm/s
86	19.3	43.3	0.48	401	4.6	1126
20	4.08	9.38	0.10	36	1.66	461
110	26.6	60.2	0.64	436	7.2	2060
87	19.2	42.9	0.50	408	3.7	1015
54	12.1	27.7	0.31	321	3.1	647
126	27.4	62.1	0.67	472	7.9	2048
45	11.1	24.6	0.28	330	1.2	204

				BPM	cm	mm/s
78	16.5	47.2	0.54	400	4.3	901
20	2.69	6.76	0.10	46	0.70	172
102	20.5	57.3	0.72	452	5.4	1085
84	15.8	46.6	0.54	405	4.4	950
43	12.9	40.2	0.44	320	3.5	610
117	21.9	60.7	0.74	493	5.7	1246
39	11.2	33.7	0.34	308	3.0	556

				BPM	cm	mm/s
98	16.6	49.2	0.48	456	2.4	533
22	6.22	4.98	0.07	24	0.65	104
141	27.8	55.5	0.56	487	3.1	648
91	16.1	49.7	0.49	458	2.3	538
84	9.9	40.2	0.38	419	1.8	396
141	29.1	59.1	0.61	505	3.7	740
54	4.2	39.2	0.35	408	1.1	326

0.90	0.89	0.37	0.36	0.45	0.50	0.62
0.37	0.24	0.04	0.04	0.52	0.50	0.39
0.30	0.35	0.19	0.95	0.01	0.01	0.01
0.11	0.97	0.57	0.24	0.03	0.00	0.00

Ao Diameter	SV	CO	VTI	Peak Vel	PA Diameter
mm	ml	ml/min	cm	mm/s	mm
1.5	0.07	30.7	3.1	687.4	1.1
0.11	0.02	9.73	0.80	123.77	0.18
1.7	0.11	46.6	4.5	877.8	1.3
1.6	0.07	26.7	2.9	658.7	1.1
1.4	0.05	22.6	2.5	540.8	0.9
1.8	0.12	50.1	4.7	934.9	1.5
1.3	0.03	11.2	1.5	439.8	0.7

mm	ml	ml/min	cm	mm/s	mm
1.5	0.08	33.9	3.1	694.1	1.1
0.14	0.03	13.48	0.52	99.76	0.15
1.7	0.16	60.6	4.0	871.8	1.5
1.5	0.07	28.1	3.1	692.7	1.1
1.4	0.05	21.2	2.4	578.5	1.0
1.8	0.15	60.8	4.1	893.6	1.4
1.3	0.02	6.9	2.0	494.6	0.8

mm	ml	ml/min	cm	mm/s	mm
1.7	0.11	42.4	2.6	618.2	1.2
0.30	0.06	23.93	0.40	94.21	0.14
2.2	0.21	87.5	3.2	719.5	1.4
1.6	0.10	34.4	2.6	628.9	1.2
1.3	0.05	18.9	2.2	460.8	1.0
2.3	0.22	90.2	3.4	806.6	1.5
1.1	-0.01	-5.5	1.8	429.8	0.9

mm	ml	ml/min	cm	mm/s	mm
1.8	0.06	27.3	2.1	544.9	1.3
0.18	0.02	11.09	0.34	94.13	0.24
2.0	0.09	44.7	2.6	638.7	1.6
1.8	0.06	24.3	2.0	564.0	1.2
1.5	0.03	15.5	1.7	407.4	1.0
2.1	0.11	49.5	2.8	733.1	1.8
1.4	0.01	5.1	1.4	356.6	0.8

0.88	0.53	0.66	0.87	0.92	0.90
0.25	0.26	0.33	0.20	0.32	0.45
0.02	0.15	0.35	0.00	0.02	0.11
0.63	0.09	0.19	0.03	0.21	0.43

SV	CO
ml	ml/min
0.0	12.7
0.01	4.12
0.0	18.0
0.0	11.2
0.0	8.7
0.1	20.9
0.0	4.4

ml	ml/min
0.0	11.9
0.01	3.79
0.1	17.9
0.0	11.6
0.0	7.4
0.1	19.4
0.0	4.3

ml	ml/min
0.0	11.7
0.01	3.15
0.0	16.5
0.0	10.1
0.0	9.1
0.0	18.0
0.0	5.4

ml	ml/min
0.0	12.2
0.01	3.56
0.0	16.2
0.0	12.3
0.0	7.9
0.0	19.3
0.0	5.1

0.92	0.72
0.80	0.66
0.58	0.86
0.62	0.79
